# First report of *Plesiochelys etalloni* and *Tropidemys langii* from the Late Jurassic of the UK and the palaeobiogeography of plesiochelyid turtles

**DOI:** 10.1098/rsos.150470

**Published:** 2016-01-13

**Authors:** Jérémy Anquetin, Sandra D. Chapman

**Affiliations:** 1 JURASSICA Museum, Route de Fontenais 21, 2900 Porrentruy, Switzerland; 2 Department of Geosciences, University of Fribourg, Chemin du Musée 6, 1700 Fribourg, Switzerland; 3 Section d’archéologie et paléontologie, Office de la culture, République et Canton du Jura, Hôtel des Halles, 2900 Porrentruy, Switzerland; 4 Department of Earth Sciences, The Natural History Museum, Cromwell Road, London SW7 5BD, UK

**Keywords:** *Plesiochelys etalloni*, *Tropidemys langii*, Plesiochelyidae, Kimmeridge Clay, Kimmeridgian, Late Jurassic

## Abstract

Plesiochelyidae is a clade of relatively large coastal marine turtles that inhabited the shallow epicontinental seas that covered western Europe during the Late Jurassic. Although the group has been reported from many deposits, the material is rarely identified at the species level. Here, we describe historical plesiochelyid material from the Kimmeridge Clay Formation of England and compare it with contemporaneous localities from the continent. An isolated basicranium is referred to the plesiochelyid *Plesiochelys etalloni* based notably on the presence of a fully ossified pila prootica. This specimen represents the largest individual known so far for this species and is characterized by remarkably robust features. It is, however, uncertain whether this represents an ontogenetic trend towards robustness in this species, some kind of specific variation (temporal, geographical or sexual), or an abnormal condition of this particular specimen. Four other specimens from the Kimmeridge Clay are referred to the plesiochelyid *Tropidemys langii*. This contradicts a recent study that failed to identify this species in this formation. This is the first time, to the best of our knowledge, that the presence of *Plesiochelys etalloni* and *Tropidemys langii* is confirmed outside the Swiss and French Jura Mountains. Our results indicate that some plesiochelyids had a wide palaeobiogeographic distribution during the Kimmeridgian.

## 1. Introduction

During the Late Jurassic, when most of Europe was covered by epicontinental seas, several groups of closely related basal eucryptodire turtles (Plesiochelyidae Baur, 1888 [1], Thalassemydidae Zittel, 1889 [2] and Eurysternidae Dollo, 1886 [3]) adapted to life at sea. These groups represent the first radiation of eucryptodire turtles into marine environments and are likely not related to modern sea turtles [4–13]. They are usually found in Kimmeridgian and Tithonian coastal marine deposits. Eurysternids are notably abundant in lagoonal paleoenvironments, such as those of Cerin and Canjuers in France, and Solnhofen in Germany. In contrast, plesiochelyids and thalassemydids are generally associated with more open shallow marine environments [6]. They have been found in great number in northwestern Switzerland, notably in the localities of Solothurn [14–17] and Porrentruy [13,18–20], but also in the region of Hannover, northwestern Germany [21–23].

Turtles have also been reported from the Late Jurassic Kimmeridge Clay Formation of southern England since the nineteenth century [24]. Many of the fragmentary remains were referred to genera (sometimes species) previously described in the Kimmeridgian of continental Europe, notably *Plesiochelys* Rütimeyer, 1873 [14], *Tropidemys* Rütimeyer, 1873 [14] and *Thalassemys* Rütimeyer, 1873 [14] (see [25]). This fragmentary material has never been properly reassessed since the end of the nineteenth century. Several more complete specimens were recognized as truly different from contemporaneous forms from continental Europe and were described as distinct taxa, notably: *Enaliochelys chelonia* Seeley, 1869 [26], *Pelobatochelys blakii* Seeley, 1875 [27] and *Tholemys passmorei* Andrews, 1921 [28]. These taxa are still considered valid today, but *Pelobatochelys blakii* was recently transferred to the genus *Tropidemys*, creating the new combination *Tropidemys blakii* [29]. Finally, recent studies confirmed the presence of two species of *Thalassemys* in the Kimmeridge Clay [20,30].

In this study, we describe several historical specimens from the Kimmeridge Clay that we refer to *Plesiochelys etalloni* (Pictet and Humbert, 1857) [31] and *Tropidemys langii* Rütimeyer, 1873 [14]. These species are otherwise known from the Kimmeridgian of the Swiss Jura Mountains [14,15,17,19]. Their presence in the British Kimmeridge Clay is therefore of major importance for the understanding of the palaeobiogeography of plesiochelyid turtles during the Late Jurassic.

## 2. Material and methods

### 2.1 Material

The first specimen described in the present paper consists of a basicranium and a fragment of right maxilla (NHMUK R3370), which we refer to *Plesiochelys etalloni* (see below). The material was collected from an unknown locality within the Kimmeridge Clay Formation and presented to the British Museum by G.E. Dibley in 1905.

The four other specimens (NHMUK OR44178b, NHMUK OR45920, NHMUK OR45921 and NHMUK R2733) described herein were all collected from the Kimmeridge Clay at Weymouth, Dorset, UK. We refer all these specimens to *Tropidemys langii* (see below). NHMUK OR44178b consists of an isolated neural bone purchased by the British Museum in 1873. This specimen was previously illustrated in Lydekker [25] and Pérez-García [29]. NHMUK OR45920 consists of the right costals 1–3 of a juvenile individual purchased by the British Museum in 1874. NHMUK OR45921 corresponds to a left first costal and was also purchased in 1874. The three aforementioned specimens were all initially referred to *Tropidemys langii* by Lydekker [25], a conclusion recently partly rejected [29]. The fourth specimen we refer to *Tropidemys langii*, NHMUK R2733, consists of a left fourth costal and was purchased by the British Museum in 1896. This specimen is therefore not mentioned in Lydekker’s catalogue [25].

### 2.2 Anatomical comparisons

The basicranium described herein (NHMUK R3370) was compared with all currently known cranial remains referred to *Plesiochelys etalloni*, namely NMB 435, NMS 8738, NMS 8739, NMS 8740, NMS 9145, NMS 40870 and NMS 40871. All of these specimens were studied first-hand by the lead author. Comparisons were also extended to other plesiochelyids for which the cranium is known: *Plesiochelys planiceps* (OUMNH J.1582), *Portlandemys mcdowelli* Gaffney, 1975 [32] (NHMUK R2914, NHMUK R3164) and *Portlandemys gracilis* Anquetin *et al.*, 2015 [13] (MJSN BSY009-708). These specimens were also studied first hand. The reader is referred to the primary literature describing these specimens [32–34] and notably to Anquetin *et al*. [13], who provided an extensive discussion of the plesiochelyid cranial anatomy. When pertinent, NHMUK R3370 was also compared with PIMUZ A/III 514, a specimen from the Tithonian of the Isle of Oléron (Department of Charente-Maritime, France) initially referred to *Thalassemys* *moseri* Bräm, 1965 [15] by Rieppel [35]. PIMUZ A/III 514 was recently designated as the holotype of a new taxon, *Jurassichelon oleronensis* Pérez-García, 2015 [36]. However, this conclusion is notably based on the spurious assertion that *Thalassemys moseri* is an invalid species name. Based on the type material from Solothurn, Anquetin *et al*. [17] have shown that *Thalassemys moseri* is indeed a valid name, albeit a referral to the genus *Thalassemys* is incorrect. Because the material from the Isle of Oléron and the specimens from Solothurn need to be properly revised, and because nomenclature is likely to change as a result, we will simply refer to this specimen by its collection number (PIMUZ A/III 514) from now on in order to avoid further confusion (see [20] for further detail). Anatomical descriptions in this study follow the nomenclature established by Gaffney [37,38] as updated by Rabi *et al*. [12].

The shell material described herein (NHMUK OR44178b, NHMUK OR45920, NHMUK OR45921 and NHMUK R2733) was notably compared with the material from Solothurn and Porrentruy that is referred to *Tropidemys langii* [17,19]. Comparisons were extended to all plesiochelyid and thalassemydid turtles known in Solothurn (see [17]) and to all the turtle material from the Kimmeridge Clay housed at the NHMUK, including *Tholemys passmorei* and *Tropidemys* (*Pelobatochelys*) *blakii*. All this material was studied first hand by the lead author. Comparisons with *Tropidemys seebachi* Portis, 1878 [39] were based on the published literature [39,40]. Anatomical descriptions of shell material follow the nomenclature established by Zangerl [41].

### 2.3 Three-dimensional model

In order to facilitate future comparisons, a three-dimensional reconstruction of the basicranium NHMUK R3370 is provided as electronic supplementary material, file S1. This model was computed with the photogrammetry software Agisoft PhotoScan v. 1.1.6 Standard Edition using sets of photographs of the specimen. We notably followed the procedures described recently by Mallison & Wings [42]. A scaled and textured high-resolution mesh (PLY format) is also available on figshare (http://dx.doi.org/10.6084/m9.figshare.1513784).

### 2.4 Institutional abbreviations

MAJ, Musée d’archéologie du Jura, Lons-le-Saunier, France; MJSN, JURASSICA Museum (formerly Musée jurassien des sciences naturelles), Porrentruy, Switzerland; NHMUK, Natural History Museum, London, UK; NMB, Naturhistorisches Museum Basel, Switzerland; NMS, Naturmuseum Solothurn, Switzerland; OUMNH, Oxford University Museum of Natural History, Oxford, UK; PIMUZ, Paläontologisches Institut und Museum, Universität Zürich, Switzerland.

## 3. Systematic palaeontology

Testudines Batsch, 1788 [43].

Eucryptodira Gaffney, 1975 [44].

Plesiochelyidae Baur, 1888 [1].

*Plesiochelys etalloni* (Pictet and Humbert, 1857) [31].

### 3.1 Holotype

MAJ 2005-11-1, a shell missing a large part of the carapace medially [31,45].

### 3.2 Type locality and horizon

‘Forêt de Lect’ near Moirans-en-Montagne, Department of Jura, France. Kimmeridgian or early Tithonian, Late Jurassic [45].

### 3.3 Referred material

**Figure 1.**
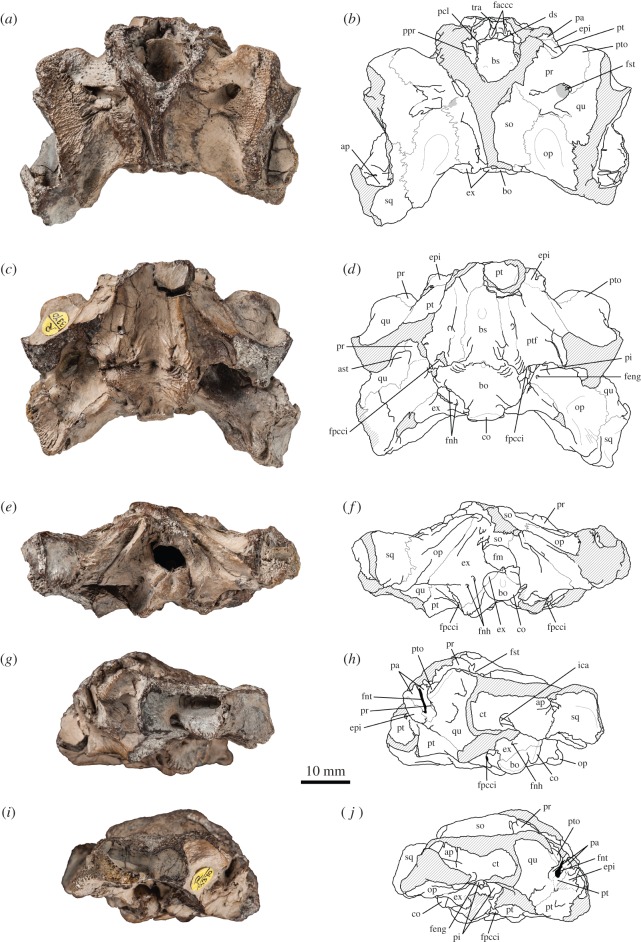
*Plesiochelys etalloni*, basicranium, NHMUK R3370, Kimmeridge Clay (unknown locality). (*a*) Photograph in dorsal view; (*b*) interpretative drawing in dorsal view; (*c*) photograph in ventral view; (*d*) interpretative drawing in ventral view; (*e*) photograph in posterior view; (*f*) interpretative drawing in posterior view; (*g*) photograph in left lateral view; (*h*) interpretative drawing in left lateral view; (*i*) photograph in right lateral view; (*j*) interpretative drawing in right lateral view. ap, antrum postoticum; ast, aditus canalis stapedio-temporalis; bo, basioccipital; bs, basisphenoid; co, condylus occipitalis; ct, cavum tympani; ds, dorsum sellae; epi, epipterygoid; ex, exoccipital; faccc, foramen anterius canalis carotici cerebralis; feng, foramen externus nervi glossopharyngei; fm, foramen magnum; fnh, foramen nervi hypoglossi; fnt, foramen nervi trigemini; fpcci, foramen posterius canalis carotici interni; fst, foramen stapedio-temporale; ica, incisura columellae auris; op, opisthotic; pa, parietal; pcl, processus clinoideus; pi, processus interfenestralis; ppr, pila prootica; pr, prootic; pt, pterygoid; ptf, pterygoid fossa; pto, processus trochlearis oticum; qu, quadrate; so, supraoccipital; sq, squamosal; tra, trabecula. Hatchings represent damaged area. Matrix is in grey.

**Figure 2.**
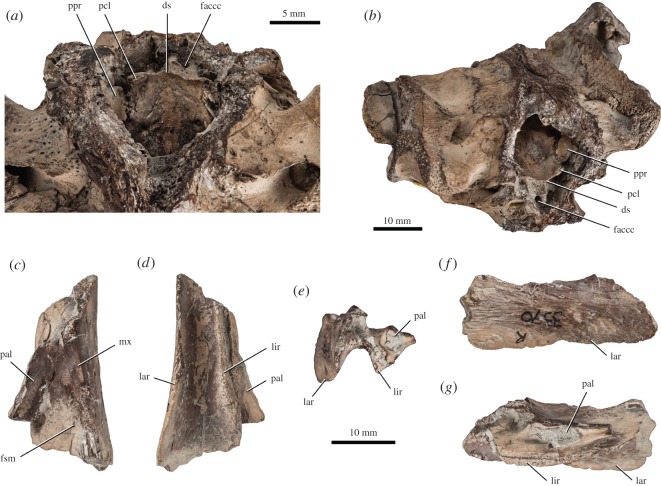
*Plesiochelys etalloni*, basicranium and maxilla, NHMUK R3370, Kimmeridge Clay (unknown locality). (*a*) Close-up of the area of the dorsum sellae in dorsal view; (*b*) anterodorsolateral view showing the left wall of the cavum cranii with the entirely ossified pila prootica; (*c*) right maxilla in dorsal view; (*d*) right maxilla in ventral view; (*e*) right maxilla in anterior view; (*f*) right maxilla in lateral view; (*g*) right maxilla in medial view. ds, dorsum sellae; faccc, foramen anterius canalis carotici cerebralis; fsm, foramen supramaxillare; lar, labial ridge; lir, lingual ridge; mx, maxilla; pal, palatine; pcl, processus clinoideus; ppr, pila prootica.

See [13,17]; NHMUK R3370, a basicranium with partial otic chambers and fragment of the right maxilla from the Kimmeridge Clay, England, UK (figures 1 and 2).

### 3.4 Occurrence

Late Jurassic (Kimmeridgian or early Tithonian) of Moirans-en-Montagne, Department of Jura, France; Late Jurassic (Kimmeridgian) of Solothurn, Canton of Solothurn and Glovelier, Canton of Jura, Switzerland; Late Jurassic (Kimmeridgian) of the Kimmeridge Clay (locality unknown), England, UK.

### 3.5 Diagnosis

See [13], reproduced here for the benefit of the reader. Differing from *Plesiochelys planiceps* in: smaller skull size; complete ossification of pila prootica; rounded foramen palatinum posterius; parietal meeting quadrate posteroventral to foramen nervi trigemini; processus trochlearis oticum moderately developed; foramen posterius canalis carotici interni on ventral surface of pterygoid; superficial canalis caroticus internus; contribution of exoccipital to condylus occipitalis minor or absent; lower lingual ridge on maxilla; lingual ridges of the lower jaw curving anteriorly in the symphyseal area. For shell characters, see [45].

### 3.6 Description and comparison

NHMUK R3370 consists of a basicranium with both otic regions (figures 1 and 2, and electronic supplementary material, file S1). The skull roof and the anterior half of the skull are missing, except a fragment of the right maxilla. The specimen is also missing large portions of the areas surrounding the cavum tympani and antrum postoticum, as well as each condylus mandibularis. As preserved, the maximal length of the basicranium is 45 mm (33 mm from the anterior fracture to the condylus occipitalis). The width of the basicranium at the base of the broken mandibular condyles is 57.3 mm. Because of its incompleteness, comparing the size of NHMUK R3370 with that of other plesiochelyid crania is difficult. However, when placed side by side with NMS 40870, one of the largest skulls known for *Plesiochelys etalloni* [13], NHMUK R3370 appears to be slightly larger. This is confirmed by unorthodox measurements, such as the midline length between the basisphenoid–pterygoid suture and the condylus occipitalis (28.4 mm compared with 25.5 mm in NMS 40870). Compared with other plesiochelyid crania, it is remarkable how robust NHMUK R3370 appears to be (electronic supplementary material, file S1). The bones seem to be proportionally thicker, but the most striking fact is that the development of every structure is generally more pronounced (see Discussion).

#### 3.6.1 Parietal

Only the posteroventralmost part of the processus inferior parietalis is preserved in NHMUK R3370 (figure 1*g*–*j*). The parietal forms at least half of the anterior margin of the foramen nervi trigemini and contacts the pterygoid ventrally, excluding the epipterygoid from that margin. On the left-hand side, the parietal forms almost all of the anterior margin of the foramen nervi trigemini. Such a variation within the same individual is known in other specimens referred to *Plesiochelys etalloni* (MNB 435, NMS 8739), but it probably has no systematic value. Posterior to the foramen nervi trigemini, the parietal sends a long process that forms all of the posterior margin of that foramen and meets the quadrate and pterygoid posteroventrally. This configuration is found in *Plesiochelys etalloni*, *Portlandemys mcdowelli*, *Portlandemys gracilis*, but also in PIMUZ A/III 514. In *Plesiochelys planiceps*, the extension of the parietal posterior to the foramen nervi trigemini does not reach the quadrate. Another interesting aspect of NHMUK R3370 resides in the fact that the foramen nervi trigemini is a slit-like opening, very much like the condition observed in most specimens referred to *Plesiochelys etalloni* (NMB 435, NMS 8738, NMS 40870, NMS 40871).

#### 3.6.2 Squamosal

The squamosal is only preserved on the left side of the specimen. The bone forms the posterolateral corner of the otic region and defines a moderately deep antrum postoticum. The development of the antrum postoticum is also moderate in *Plesiochelys etalloni* (NMB 435, NMS 8739), *Plesiochelys planiceps* and PIMUZ A/III 514. In contrast, this structure is slightly less developed in *Portlandemys gracilis*. The condition in *Portlandemys mcdowelli* is unknown. Posterior to the cavum tympani, the lateral surface of the squamosal forms a wide concavity. The anterodorsal and posterolateral parts of the squamosal are broken in NHMUK R3370. However, the posteroventral extension of the squamosal along the processus paroccipitalis of the opisthotic is preserved. There, the squamosal forms a rugose area for the attachment of the M. depressor mandibulae [46]. As preserved, the bone contacts the quadrate anteriorly and anteroventrally, and the opisthotic medially. The suture with the quadrate is poorly preserved within the antrum postoticum.

#### 3.6.3 Maxilla

Associated with the basicranium is an isolated fragment of the right maxilla (figure 2*c*–*g*). This fragment corresponds to the middle part of the maxilla. The anterodorsal process that contacts the prefrontal, the premaxilla and the nasal, and the posterodorsal process that meets the jugal are missing. This fragment therefore mainly consists of the triturating surface ventrally and the middle part of the orbital floor dorsally. A small fragment of the right palatine is still sutured to the maxilla dorsomedially. The lingual ridge is broad and well developed, and increases in height anteriorly. The palatine does not contribute to the lingual ridge. The labial ridge is slender and very high. The trough between the labial and lingual ridges is deep. This description matches the condition observed in *Plesiochelys etalloni* and *Plesiochelys planiceps*. However, it should be noted that the lateral surface of the maxilla is gently concave. This condition is also found in NMS 40870, a large, subcomplete skull referred to *Plesiochelys etalloni* [13]. Part of the sutural surface with the jugal is still visible in the posterolateral corner of the dorsal surface of the maxilla. Anteromedial to this area, the maxilla is pierced by the foramen supramaxillare.

#### 3.6.4 Quadrate

The quadrate is equally preserved on both sides. The condyli mandibularis and the margins of the cavum tympani are missing. The contacts of the quadrate are the same as in other plesiochelyids: squamosal posterolaterally, opisthotic posteromedially, prootic anteromedially and pterygoid anteroventrally. In contrast to *Plesiochelys planiceps*, the quadrate also meets the parietal posteroventral to the foramen nervi trigemini. As usual, the quadrate forms half of the foramen stapedio-temporale and a well-developed cavum tympani. The incisura columellae auris remains open posteroventrally. Because the processus articularis of the quadrate is broken on each side, the presence of the ventrally infolding ridge, so typical of plesiochelyid and eurysternid turtles [13], cannot be confirmed in NHMUK R3370. The processus trochlearis oticum is strongly developed in NHMUK R3370 and recalls the condition in *Plesiochelys planiceps* and *Portlandemys mcdowelli*. In other plesiochelyids, notably in other specimens referred to *Plesiochelys etalloni*, the processus trochlearis oticum is usually less robust. However, as noted above, NHMUK R3370 is characterized by the robustness of all of its structures (see Discussion). The dorsal surface of the quadrate posterior to the processus trochlearis oticum is sculptured with many low ridges and pits, which are absent in other plesiochelyid crania.

#### 3.6.5 Epipterygoid

Only the posterior half of the epipterygoid is preserved on each side of specimen NHMUK R3370 (figure 1*g*–*j*). The sutures in the ethmoid region are rather difficult to see, because the basicranium is broken at this level. The epipterygoid contacts the parietal dorsally and the pterygoid ventrally and posteriorly. It is excluded from the anterior margin of the foramen nervi trigemini by a contact between the parietal and pterygoid. As aforementioned, this configuration is common to all plesiochelyids and is also found in PIMUZ A/III 514. The posteroventral corner of the epipterygoid is damaged on each side. It is therefore difficult to assess whether or not the fossa cartilaginis epipterygoidei remained open in that specimen. A small space might have been present on the right-hand side, but apparently not on the left-hand side. Because of the poor preservation, nothing can be said about the contacts on the medial surface of the epipterygoid.

#### 3.6.6 Pterygoid

The anterior part of the pterygoid where the bone contacts the palatine and extends laterally to form the processus pterygoideus externus is missing in NHMUK R3370. The pterygoids are broken a few millimetres in front of the anterior tip of the basisphenoid as seen in ventral view. The part of the pterygoid that extends posterolaterally along the mandibular process of the quadrate is also broken on each side. As preserved, the pterygoid contacts the basioccipital posteromedially, the basisphenoid medially, the other pterygoid anteromedially and the quadrate antero- and laterodorsally. In the ethmoid region, the pterygoid also contacts the epipterygoid and the parietal (see above). The pterygoid forms the ventral margin of the foramen nervi trigemini. Anteromedially, the floor of the cavum epiptericum where the pterygoid usually forms the foramen anterius canalis carotici palatinum and foramen nervi vidiani is not preserved in NHMUK R3370. Posteriorly, the pterygoid floors the cavum acustico-jugulare. In this region, the pterygoid contacts the processus interfenestralis of the opisthotic, the basioccipital and the exoccipital.

The two ridges that define the pterygoid fossa on the ventral surface of the pterygoid are very pronounced in NHMUK R3370. The pterygoid fossa is deep, but it is not bordered posteriorly by a ridge as in *Portlandemys mcdowelli*. The ridge defining the medial border of the pterygoid fossa is comparatively lower and less developed in *Plesiochelys planiceps* and *Portlandemys mcdowelli*. The high and acute ridge observed in NHMUK R3370 recalls the condition in NMS 40870 (a large subcomplete skull referred to *Plesiochelys etalloni*) and MJSN BSY009-708 (the holotype of *Portlandemys gracilis*). However, in contrast to NMS 40870 and most other crania referred to *Plesiochelys etalloni*, the canalis caroticus internus is not open ventrally anteromedial to that ridge in NHMUK R3370. NMB 435, a juvenile individual, is the only other specimen referred to *Plesiochelys etalloni* in which the canalis caroticus internus is completely floored along the basisphenoid–pterygoid suture. The ventral surface of the pterygoid in NMB 435, however, lacks pronounced ridges, and the pterygoid fossa is relatively shallow. In NHMUK R3370, a pair of foramina occurs along the medial ridge of the pterygoid fossa. These foramina cannot be probed, but it is likely that they communicate with the canalis caroticus internus. Gaffney [44] discussed a similar pair of foramina in the eurysternid *Solnhofia parsonsi* Gaffney, 1975 [44]. They are also present in the eurysternid *Parachelys eichstaettensis* Meyer, 1864 [47] (NHMUK OR42888). Finally, a smaller, but similarly placed foramen occurs on the left-hand side of the holotype of *Plesiochelys planiceps* (OUMNH J.1582), although it is not described in the literature. The nature of these foramina, either in *Solnhofia parsonsi*, *Parachelys eichstaettensis*, *Plesiochelys planiceps* or in NHMUK R3370, remains uncertain. Several parallel, short ridges occur on the posteromedial flange of the pterygoid, where the bone meets the basioccipital. Similar ridges also occur close by on the basisphenoid and basioccipital. The foramen posterius canalis carotici interni occurs at the posterior margin of the pterygoid and is formed entirely by that bone. As such, the foramen posterius canalis carotici interni is barely apparent in ventral view (notably on the left-hand side where the margin of the foramen is not broken), which recalls the condition in *Plesiochelys planiceps* and *Portlandemys gracilis*.

#### 3.6.7 Supraoccipital

The anterodorsal part of the supraoccipital, as well as the crista supraoccipitalis are missing in NHMUK R3370. A long lengthwise fracture occurs on the left part of the supraoccipital. Medial to this fracture, the roof of the cavum cranii caved in. As preserved, the supraoccipital contacts the prootic anterolaterally, the opisthotic posterolaterally and the exoccipital posteroventrally. The lateral corner of the supraoccipital, where the bone meets the prootic and the opisthotic, is sculptured with a few short ridges (especially visible on the left-hand side). A short, arched ridge is also present in this area in NMS 40870, a specimen referred to *Plesiochelys etalloni* [13].

#### 3.6.8 Exoccipital

As in most turtles, the exoccipital forms the lateral margin of the foramen magnum and contacts the supraoccipital dorsomedially, the opisthotic dorsolaterally and the basioccipital ventromedially. In NHMUK R3370, the exoccipital also extends anteroventrally and meets the pterygoid ventral to the processus interfenestralis. This contact between the exoccipital and pterygoid is also present in *Portlandemys mcdowelli*, *Portlandemys gracilis* (probably) and *Plesiochelys etalloni*. This anteroventral extension of the exoccipital also probably contacted the processus interfenestralis, but this process is broken on each side in NHMUK R3370. The exoccipital forms a small dorsolateral part of the condylus occipitalis. However, the exoccipitals do not meet one another, either on the condylus occipitalis or more anteriorly in the floor of the cavum cranii. In *Plesiochelys planiceps*, the exoccipital forms one-third of the condylus occipitalis and meets the other exoccipital on the condyle and more anteriorly preventing the exposure of the basioccipital in the ventral margin of the foramen magnum. In *Portlandemys mcdowelli* (at least in NHMUK R3164), the exoccipital forms a smaller portion of the condylus occipitalis (as in NHMUK R3370) and meets its counterpart only more anteriorly where they prevent the exposure of the basioccipital in the ventral margin of the foramen magnum. Gaffney [34] described the condition in *Plesiochelys etalloni* as identical to that of *Portlandemys mcdowelli*. However, observation of the material reveals that it is not the case. In NMS 8738 and NMS 8739, the exoccipital–basioccipital suture is mostly obliterated preventing any confident conclusion. In NMS 9145, the exoccipital participates only to the neck of the condylus occipitalis and does not meet the other exoccipital in the ventral margin of the foramen magnum. A similar arrangement is present in NMS 40870, although the exoccipitals do meet in the midline more anteriorly in the floor of the cavum cranii [13]. However, the basioccipital is still exposed on the ventral margin of the foramen magnum in that specimen. Therefore, the condition in NHMUK R3370 is closer to that observed in *Plesiochelys etalloni*, although the small contribution of the exoccipital to the condylus occipitalis is a notable difference.

A ridge occurs on the posterior surface of the exoccipital in many turtles. This ridge is generally subparallel to the opisthotic–exoccipital suture. As in *Plesiochelys etalloni* and *Plesiochelys planiceps*, this ridge is usually low and rounded. In contrast, this ridge is more pronounced and acute in *Portlandemys mcdowelli* (area poorly preserved in *Portlandemys gracilis*). In NHMUK R3370, this ridge is very pronounced and acute. But again, it should be noted that this cranium is characterized by a strong development of many of its structures (see Discussion).

#### 3.6.9 Basioccipital

The basioccipital contacts the basisphenoid anteriorly, the pterygoid anterolaterally and the exoccipital dorsolaterally. The basioccipital forms most of the condylus occipitalis and, as noted above, is exposed in the ventral margin of the foramen magnum. Inside the cavum cranii, the dorsal surface of the basioccipital is heavily sculptured. A sagittal ridge extends from the greatly developed basis tuberculi basalis almost up to the condylus occipitalis posteriorly. The height of this ridge decreases posteriorly. Two pairs of ridges also extend posterolaterally from the basis tuberculi basalis and probably reach the exoccipitals laterally. Such sculpturing of the dorsal surface of the basioccipital is not known in other plesiochelyids, but it should be noted that this area is rarely as well preserved as in NHMUK R3370. On the ventral surface of the basioccipital, the tubercula basioccipitale are greatly developed and extend ventrolaterally from the basicranium. Owing to the great development of the tubercula, a large depression occurs in the midline between these structures. The bone surface on the crest of each tuberculum basioccipitale is rugose. Close to the suture with the pterygoid, the basioccipital bears several short ridges like the ones found nearby on the pterygoid and basisphenoid. A deep pit occurs in the midline along the basioccipital–basisphenoid suture. All of these structures (tubercula basioccipitale, ridges and pit) stand out as overly developed when compared with other plesiochelyids.

#### 3.6.10 Prootic

The anteromedial part of the prootic is damaged on each side of NHMUK R3370. On the dorsal surface of the otic chamber, the bone contacts the supraoccipital posteromedially, the opisthotic posteriorly and the quadrate laterally. The posterior contact with the opisthotic is relatively broad and clearly prevents the supraoccipital and quadrate from meeting posteromedial to the foramen stapedio-temporale. In this area, the prootic produces several well-developed crest-like structures. The prootic also forms similar structures on the opposite side of the sulcus left by the stapedial artery on the dorsal surface of the otic chamber. In other plesiochelyids, the sulcus of the stapedial artery is simply bordered by low ridges that rapidly decrease in height as the artery winds away from the foramen stapedio-temporale. Anterodorsally, the prootic forms about one-third of the robust processus trochlearis oticum. In dorsal view, the prootic and quadrate contribute in equal proportions to the processus trochlearis oticum, but the contribution of the prootic is greatly reduced in anteroventral view. Ventromedial to the processus trochlearis oticum, the prootic contacts the parietal and quadrate and is excluded from the posterior margin of the foramen nervi trigemini in lateral view by a contact between these two bones. The medial surface of the prootic inside the cavum cranii is poorly preserved, but the flat sheet of bone formed by the ossified pila prootica is preserved on the left-hand side and is still articulated with the processus clinoideus of the basisphenoid (figure 2*a*,*b*). This is a very important character since among plesiochelyids the pila prootica is entirely ossified only in *Plesiochelys etalloni* [13].

#### 3.6.11 Opisthotic

The posterolateral part of the right opisthotic is damaged in NHMUK R3370, but the left opisthotic is complete. The bone contacts the prootic anteriorly, the quadrate anterolaterally, the squamosal posterolaterally, the exoccipital posteroventromedially and the supraoccipital medially. The dorsal surface of the opisthotic forms a large and deep concavity. This concavity is bordered posteriorly by a pronounced ridge formed by the posterior margin of the opisthotic. Both this concavity and this ridge are absent in other known plesiochelyids. A ridge is also present on the posteroventral margin of the processus paroccipitalis. The posterolateral part of the ventral surface of the opisthotic is sculptured with short ridges that probably served for muscular attachment. The distal half of the processus interfenestralis of the opisthotic is broken on each side, but the broken part is still sutured to the pterygoid, basioccipital and exoccipital.

#### 3.6.12 Basisphenoid

In ventral view, the basisphenoid is a triangular to ogival (forming a pointed arch) element that contacts the pterygoid anteriorly and laterally and the basioccipital posteriorly (figure 1*c*,*d*). The way the ventromedial fold of the pterygoid reduces the lateral exposure (and therefore the apparent width) of the basisphenoid is particularly visible in that specimen. The ventral surface of the basisphenoid is slightly concave, but the overall concavity of this region is greatly increased by the pronounced parasagittal ridge that occurs on the pterygoid. A small, oval-shaped depression occurs in the midline on the anterior part of the basisphenoid. Similar shallow depressions (paired or single) exist in other parts of the basisphenoid in *Plesiochelys planiceps*, *Portlandemys mcdowelli* and *Portlandemys gracilis*. Several oblique ridges are present in the posterolateral corners of the basisphenoid in NHMUK R3370. They mirror those present on the pterygoid and basioccipital in the same region. As mentioned above, a deep median pit occurs on the basisphenoid–basioccipital suture. These ridges and this pit are not found in other plesiochelyids.

Inside the cavum cranii, the dorsum sellae is high and does not overhang the sella turcica (figure 2*a*,*b*). As noted above, the processus clinoideus is sutured to a completely ossified, sheet-like pila prootica. The surface below the dorsum sella is mostly vertical, so that the foramen anterius canalis carotici cerebralis opens only a short distance in front of the level of the dorsum sellae. The sagittal ridge on this surface is well developed. The bar of bone that separates the foramina anterius canalis carotici cerebralis is about the same width as one of these foramina. The basicranium is broken anterior to the foramina anterius canalis carotici cerebralis. Only the anterior tip of the left trabecula can be confidently identified, but it is not in anatomical position. The mostly vertical surface below the dorsum sellae corresponds to the condition found in *Plesiochelys etalloni* and *Plesiochelys planiceps*, and it is clearly different from the long, gently sloping surface present in *Portlandemys mcdowelli* and *Portlandemys gracilis* (see [13]). However, the completely ossified pila prootica is found only in *Plesiochelys etalloni*.

*Tropidemys langii* Rütimeyer, 1873 [14].

### 3.7 Lectotype

NMS 16, the posterior part of a carapace [15,17].

### 3.8 Type locality and horizon

Solothurn, Canton of Solothurn, Switzerland. Solothurn Turtle Limestone, uppermost member of the Reuchenette Formation, late Kimmeridgian, Late Jurassic [16,48].

### 3.9 Referred material

**Figure 3.**
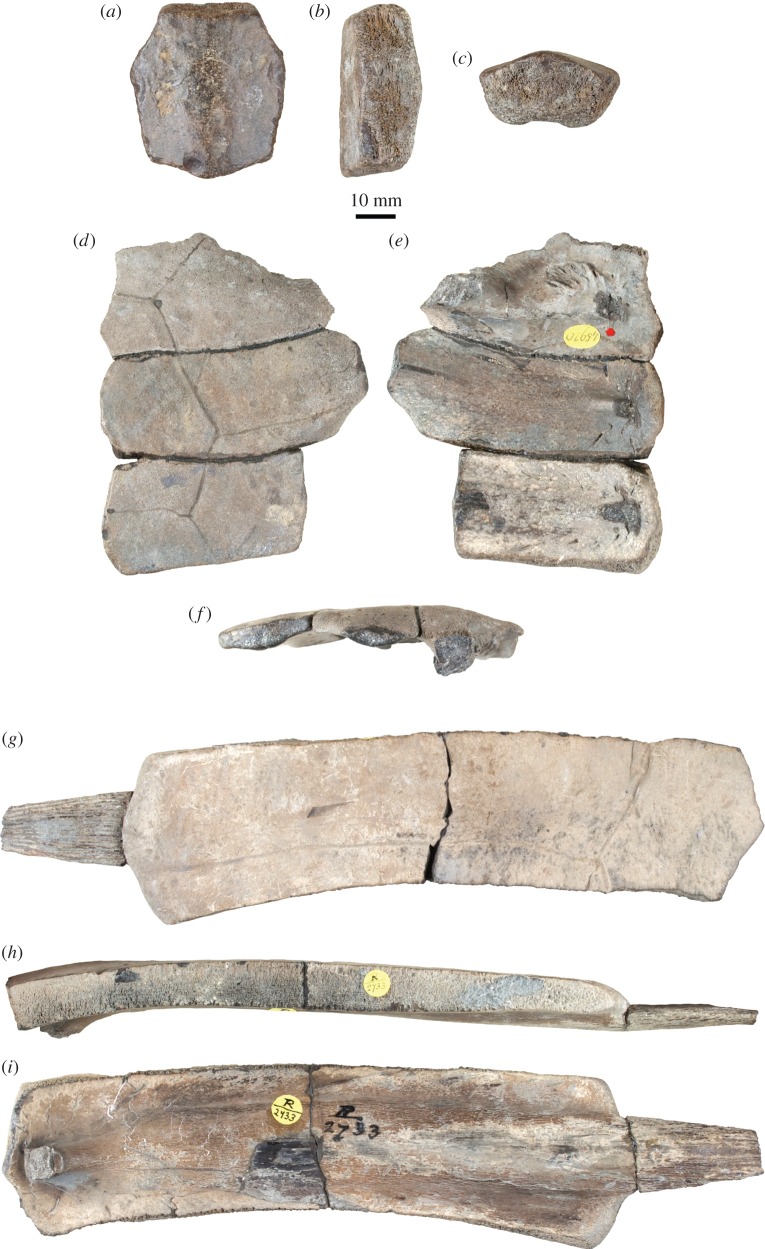
*Tropidemys langii*, Kimmeridge Clay of Weymouth, Dorset, UK. (*a*–*c*) NHMUK OR44178b, isolated (fourth?) neural bone in dorsal (*a*), right lateral (*b*) and anterior (*c*) views; (*d*–*f*) NHMUK OR45920, right costals 1–3 of a juvenile in dorsal (*d*), ventral (*e*) and lateral (*f*) views; (*g*–*i*) NHMUK R2733, large left costal 4 in dorsal (*g*), anterior (*h*) and ventral (*i*) views.

**Figure 4.**
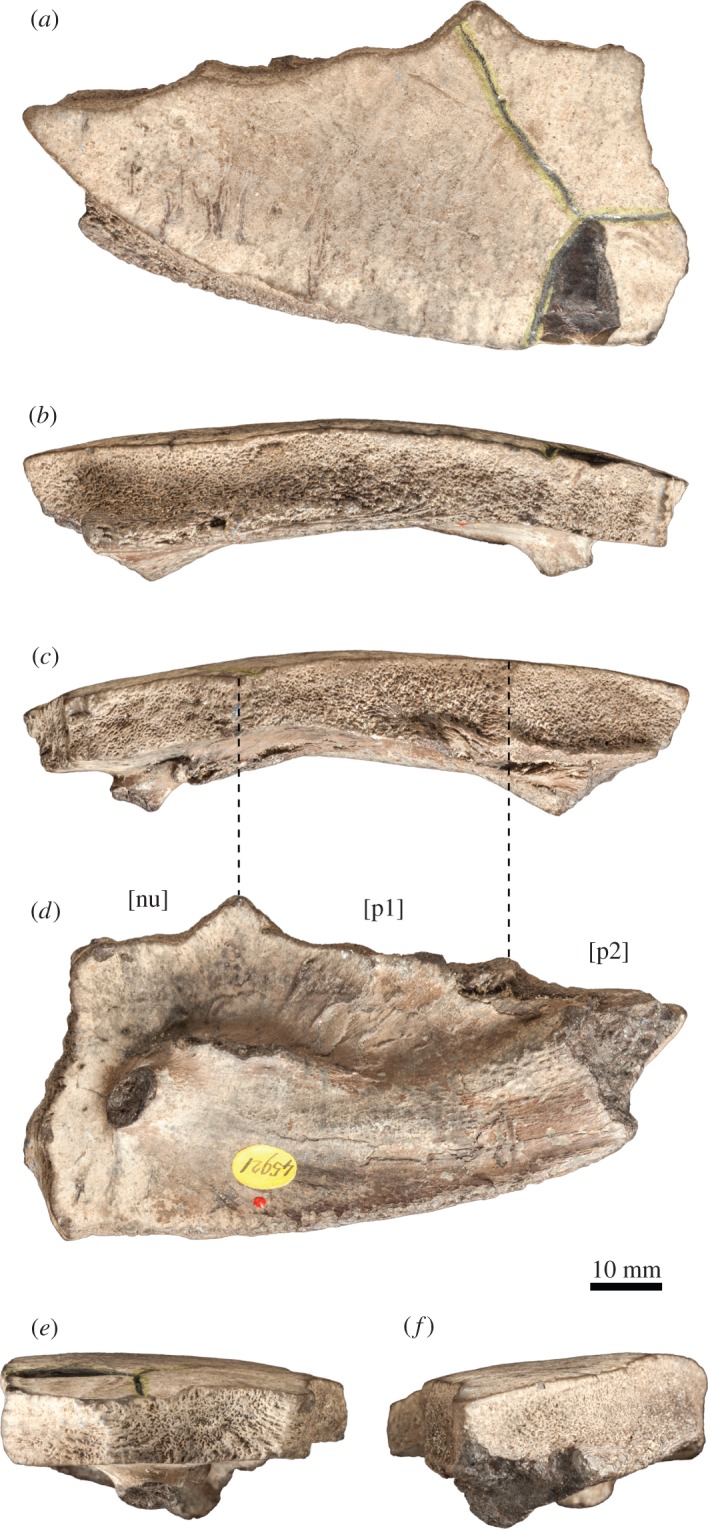
*Tropidemys langii*, isolated left first costal, NHMUK OR45921, Kimmeridge Clay of Weymouth, Dorset, UK. (*a*) dorsal view; (*b*) posterior view; (*c*) anterior view; (*d*) ventral view; (*e*) medial view; (*f*) lateral view. [nu], surface of suture with nuchal; [p1] surface of suture with peripheral 1; [p2] surface of suture with peripheral 2.

See [19]. Specimens from the Kimmeridge Clay of Weymouth, Dorset, UK: NHMUK OR44178b, an isolated neural; NHMUK OR45920, right costals 1–3; NHMUK OR45921, a left first costal; NHMUK R2733, a left fourth costal (figures 3 and 4).

### 3.10 Occurrence

Late Jurassic (late Kimmeridgian) of Solothurn, Canton of Solothurn, Switzerland; Late Jurassic (early Kimmeridgian) of Porrentruy, Canton of Jura, Switzerland; Late Jurassic (Kimmeridgian) of Weymouth, Dorset, UK.

### 3.11 Diagnosis

See [19].

### 3.12 Description and comparison

NHMUK OR44178b consists of a large isolated neural bone (length=43 mm; width=39.6 mm; figure 3*a*–*c*). The dorsal surface of the neural is tectiforme, hence forming a sagittal keel. The bone is remarkably thick (19 mm medially). Anteriorly, the contact with the preceding neural is moderately concave. Posteriorly, the contact with the following neural is convex and formed by two oblique facets, one on each side of the sagittal keel. The anterolateral margin of the neural is elongated, representing about 75% of the length of the posterolateral margin. These proportions, the absence of scale sulcus and the tectiforme dorsal surface are more consistent with an identification as a fourth neural plate. This specimen was figured by Lydekker [25] and identified as a second neural of *Tropidemys langii*. In a recent review, Pérez-García [29] considered this neural to be either a second or fourth neural plate and cautiously referred it to *Tropidemys* sp. The latter author correctly pointed out that a referral to *Tropidemys seebachi* or *Tropidemys* (*Pelobatochelys*) *blakii* could be excluded. The absence of intervertebral sulcus on NHMUK OR44178b is incompatible with the peculiar morphology exhibited by *Tropidemys seebachi* [40], whereas the relative width of the neural excludes a referral to *Tropidemys* (*Pelobatochelys*) *blakii* [29]. However, Pérez-García [29] refrained from referring this large neural to *Tropidemys langii*, the only other species in the genus, notably because no other specimens referable to this species were known in the UK. In this we disagree, because at least three other specimens from the Kimmeridge Clay at Weymouth can be confidently assigned to *Tropidemys langii* (see below). Therefore, based on the size and morphological characteristics of this isolated neural and on the presence of other specimens clearly referable to *Tropidemys langii* in the same locality, we feel it is fairly safe to refer NHMUK OR44178b also to this species.

NHMUK OR45920 consists of the right first to third costals of a juvenile individual (figure 3*d*–*f*). The first costal (length=59.4 mm; medial width=27.3 mm) is complete, but the distal part of the second (length=70 mm; medial width=24.8 mm) and third costals is damaged. The thickness of the bones is remarkable. Anteriorly, the first costal had a sutural contact with the nuchal and the first peripheral, but the contact with the second costal was not sutural, and a fontanelle may have been present. There the distal tip of the second thoracic rib protrudes from the costal. The contacts between the second costal and peripherals 3 and 4 were also non-sutural. The posterior margin of the first costal is markedly convex distally. On the ventral surface of the first costal, there is a strong articulation site for the first thoracic rib. Medially, the contacts of the second and third costals with adjoining neurals reveal that the anterolateral margin of these neurals was elongated. This description is fairly consistent with *Tropidemys langii*, and the pattern of vertebral scales clearly excludes *Tropidemys seebachi* and *Tropidemys* (*Pelobatochelys*) *blakii*. As a matter of fact, NHMUK OR45920 can be directly compared with MJSN CRE985-1, a slightly more advanced juvenile specimen of *Tropidemys langii* from the early Kimmeridgian of Porrentruy, Switzerland [19].

NHMUK OR45921 consists of an isolated left first costal of a larger individual (length=96 mm; medial width=42.7 mm; figure 4). The bone is very thick. There are no fontanelles between the costal and adjoining peripherals. The posterior margin of the costal is remarkably convex distally. Once again, these characteristics and the pattern of scale sulci are consistent with *Tropidemys langii* and exclude de facto *Tropidemys seebachi* and *Tropidemys* (*Pelobatochelys*) *blakii*. On the ventral surface of the costal, a well-developed, crest-like articular site for the first thoracic rib is present. This structure extends laterally and appears to be continuous with the axillary buttress. This morphology was described in *Tropidemys langii* by Püntener *et al*. [19], but the condition in *Tropidemys seebachi* is unknown. *Tropidemys* (*Pelobatochelys*) *blakii* lacks this morphology (see NHMUK R2).

The two aforementioned specimens, NHMUK OR45920 and NHMUK OR45921, were initially referred to *Tropidemys langii* by Lydekker [25], a conclusion rejected by Pérez-García [29] without further explanation. However, the carapace morphology of *Tropidemys langii* is now very well known [19], and these two specimens can be confidently assigned to this species.

NHMUK R2733 is a large left costal (length=165 mm without rib tip; medial width=40 mm; figure 3*g*–*i*). The costal is slightly arched posteriorly and slightly wider distally than proximally. A strong conical rib tip protrudes from the distal margin of the costal plate. The costal remains thick up to the distal margin, but the latter is rounded as seen in transverse section. Therefore, there was no strong sutural contact between this costal and adjoining peripherals, although costo-peripheral fontanelles were probably absent. Based on our own experience of the material from Porrentruy described by Püntener *et al*. [19], a strong sutural contact between most costals and peripherals was probably established late during ontogeny in *Tropidemys langii*. Medially, the two sutural contacts with adjoining neurals are of similar length, which means that the neurals had elongated anterolateral margins. Two vertebro-pleural and one interpleural sulci are apparent on the dorsal surface of the costal. The vertebral scale was narrow, whereas the two pleurals were much wider. Based on the above description, NHMUK R2733 can be safely identified as a left fourth costal of *Tropidemys langii*. This specimen was purchased by the British Museum in 1896 and therefore is not mentioned in Lydekker [25].

## 4. Discussion

### 4.1 Taxonomic assignment of the basicranium NHMUK R3370

NHMUK R3370 is notably characterized by a high dorsum sellae that does not overhang the sella turcica. This remarkable configuration is known only in a group of Late Jurassic turtles (*Plesiochelys etalloni*, *Plesiochelys planiceps*, *Portlandemys mcdowelli* and *Portlandemys gracilis*) traditionally referred to the Plesiochelyidae and in Pan-Chelonioidea Joyce *et al*. [49], which appear much later during the Early Cretaceous [13,34]. Among plesiochelyids, the species referred to the genus *Portlandemys* Gaffney, 1975 [32] exhibit a long and gently sloping surface below the dorsum sellae. As a result, the foramina anterius canalis carotici cerebralis open a long distance in front of the level of the dorsum sellae in these species [13]. In contrast, the surface below the dorsum sellae is mostly vertical in *Plesiochelys etalloni* and *Plesiochelys planiceps*, which results in foramina anterius canalis carotici cerebralis opening only a short distance in front of the level of the dorsum sellae. This is the condition present in NHMUK R3370.

A referral of NHMUK R3370 to *Plesiochelys planiceps* is however contradicted by several diagnostic features. First, the holotype of *Plesiochelys planiceps* (OUMNH J.1582) is at least as large as NHMUK R3370, but most of its cranial sutures are still open, indicating that the specimen had not reached the adult stage at the time of death. The sutures of NHMUK R3370 are all tightly closed. Second, in *Plesiochelys planiceps*, the process of the parietal posterior to the foramen nervi trigemini does not reach the quadrate as it does in other plesiochelyids and NHMUK R3370. Third, *Plesiochelys planiceps*, like all other plesiochelyids except *Plesiochelys etalloni*, lacks a completely ossified pila prootica. The presence of an entirely ossified pila prootica in NHMUK R3370 is actually the strongest argument in favour of a referral of this specimen to *Plesiochelys etalloni*. The concave lateral surface of the maxilla and the slit-like foramen nervi trigemini are other arguments pointing in the same direction.

As pointed out in the above description, a number of characteristics distinguish NHMUK R3370 from other specimens referred to *Plesiochelys etalloni*, as well as other plesiochelyids. Most of these differences seem, however, to be linked with the increased robustness of this specimen: strongly developed processus trochlearis oticum; acute ridges on the exoccipital and opisthotic; deep concavity on the dorsal surface of the opisthotic; crest-like structures on the posterior part of the prootic; strong development of the diverse structures (ridges, scars for muscular attachment, cavities) on the ventral surface of the basicranium; pterygoid fold slightly more developed posteriorly, resulting in a foramen posterius canalis carotici interni opening closer to the posterior margin of the pterygoid. These put aside, the remaining differences between NHMUK R3370 and the specimens referred to *Plesiochelys etalloni* are relatively limited. The canalis carotici interni is completely floored by the pterygoid in NHMUK R3370, whereas its anterior half remains open in most specimens referred to *Plesiochelys etalloni*. However, the canalis carotici interni is also entirely floored in NMB 435, one of the smallest crania known for *Plesiochelys etalloni*. The exoccipital contributes to the formation of the condylus occipitalis in NHMUK R3370, whereas in other specimens referred to *Plesiochelys etalloni* the condylus is formed by the basioccipital only. However, the contribution of the exoccipital in NHMUK R3370 is very limited and could result from the overall increased robustness observed in that specimen. Finally, the last major difference between NHMUK R3370 and other specimens referred to *Plesiochelys etalloni* is the presence of a pair of foramina on the ridge forming the medial border of the pterygoid fossa. As noted above (see §3.6.6), similar foramina occur in *Plesiochelys planiceps* (on the left-hand side only) and in the eurysternids *Solnhofia parsonsi* and *Parachelys eichstaettensis*. Gaffney [44] considered these foramina to be the posterior opening of the canalis carotici palatinum in *Solnhofia parsonsi*, but this would involve an important modification in the architecture of the internal carotid artery. The nature, homology and systematic value of these foramina therefore remain to be confirmed.

In conclusion, putting aside the general robustness of the specimen and the presence of this additional pair of foramina on the pterygoid, the morphology of NHMUK R3370 is consistent with a referral to *Plesiochelys etalloni*. The best arguments in favour of this conclusion are the concave lateral surface of the maxilla, the slit-like foramen nervi trigemini, the configuration of the dorsum sellae and sella turcica, and the presence of a completely ossified pila prootica. Although this is not in itself a compelling argument, it should also be noted that NHMUK R3370 and most other specimens referred to *Plesiochelys etalloni* (the age of the holotype is somewhat ambiguous, see [45]) are dated from the Kimmeridgian.

### 4.2 Old, variant or abnormal?

As discussed above, NHMUK R3370 is best interpreted as a large specimen of *Plesiochelys etalloni*. However, this cranium is characterized by a set of features previously not known in this species, or, for most of them, in any other plesiochelyid. Many osseous structures of the cranium seem overly developed or exaggerated in this specimen, leading to an increased robustness.

Because NHMUK R3370 is now the largest specimen referred to *Plesiochelys etalloni*, it is reasonable to assume that it is also the specimen with the most advanced ontogenetic stage. NMB 435 and NMS 8738 are the smallest crania referred to *Plesiochelys etalloni*. Although they are already relatively large individuals (skull length around 60 mm), many of their sutures are still open. In these specimens, the ventral surface of the basicranium is relatively flat, and the ridges bordering the pterygoid fossa are low. The bone surface lacks any particular sculpturing. In addition, the lateral surface of the maxilla is straight. In NMS 40870 (a larger specimen with a skull length of around 80 mm), the ridges bordering the pterygoid fossa are more pronounced than in NMB 435 and NMS 8738, and the medial area on the basisphenoid and the lateral surface of the maxilla are concave. Finally, in NHMUK R3370, the lateral surface of the maxilla is also concave, the ridges bordering the pterygoid fossa are even more pronounced, and numerous additional ridges and scars for muscular attachment have appeared in many parts of the cranium. This sequence may indicate that late ontogenetic stages in *Plesiochelys etalloni* are characterized by an increased robustness without much increase in size, because NHMUK R3370 is only slightly larger than NMS 40870. However tempting this hypothesis is, testing it would require additional material. Increased robustness during ontogeny has already been signalled in the plesiochelyid *Portlandemys mcdowelli* [13].

Of course, the above is only one of many possible explanations for the data. NHMUK R3370 may be an abnormal individual, suffering some condition that led to an increased robustness of the skeleton. This robustness may also be the result of temporal and/or geographical variations. After all, all other crania referred to *Plesiochelys etalloni* come from the late Kimmeridgian of Switzerland, and all but one from the same locality (Solothurn). Sexual dimorphism may also be a plausible explanation. For the time being, we have no argument to either confirm or refute any of the above hypotheses.

### 4.3 Turtle material from the Kimmeridge Clay

Three taxa have been named based on material from the Kimmeridge Clay and remain for the moment unique to that formation: *Enaliochelys chelonia*, *Pelobatochelys blakii* and *Tholemys passmorei*. Following Lydekker [25], *Enaliochelys chelonia* was long considered a synonym of *Thalassemys hugii* Rütimeyer, 1873 [14], but a recent revision of the material was able to show that this taxon is indeed valid [36]. *Enaliochelys chelonia* is only known from the Kimmeridge Clay at Ely, Cambridgeshire, UK. Its relationships with other Late Jurassic coastal marine turtles remain obscure. *Pelobatochelys blakii* is a peculiar turtle characterized by the presence of a medial keel on neurals and poorly ossified costals with the rib apparent on the dorsal surface of the plate distally. This taxon was mostly overlooked since its initial description [27], but was considered valid by Lydekker [25], Delair [50] and Kuhn [51]. Based on the presence of keeled neurals, Püntener *et al*. [19] tentatively suggested that this taxon should be referred to *Tropidemys* sp., but concluded that the material was too incomplete to provide a definitive diagnosis. Pérez-García [29] recently revised the material and proposed the new combination *Tropidemys blakii*. However, several characteristics appear to contradict a referral of this species to *Tropidemys*: elongated neurals with shorter anterolateral margins, reduced ossification of costals, and very wide vertebral scales. *Tropidemys* (*Pelobatochelys*) *blakii* is only known from the Kimmeridge Clay at Weymouth, Dorset, UK. Finally, *Tholemys* *passmorei* is based on a single complete shell from the Kimmeridge Clay at Swindon, Wiltshire, UK. This taxon was briefly discussed by Lapparent de Broin *et al*. [6] and included in some phylogenetic analyses [52–54], but its relationships remain elusive.

The rest of the turtle fauna from the Kimmeridge Clay housed at the NHMUK mainly consists of disarticulated shell elements and limb bones and was mostly overlooked during the twentieth century. Most of this material remains indeterminable: two isolated cervical vertebra (NHMUK R3371); three isolated neural plates (NHMUK OR44178x, NHMUK OR44178y and NMHUK OR44178z) tentatively referred to *Plesiochelys*? sp. by Lydekker [25]; a collection of isolated humeri and femora (NHMUK OR43033, NHMUK OR43570, NHMUK OR44180, NHMUK OR44180a, NHMUK OR44180b and NHMUK OR45923); a small costal plate ornamented with strong radiating ridges (NHMUK OR41403); a hyoplastron, possibly belonging to an immature individual (NHMUK OR42372), incorrectly referred to *Tropidemys langii* by Lydekker [25] (see [29]); shell fragments (NHMUK OR46326 and NHMUK OR46327); and a femur (NHMUK OR46328) tentatively referred to *Thalassemys hugii* Rütimeyer, 1873 [14] by Lydekker [25], but deemed undiagnostic by Pérez-García [36]. One specimen from the Etches Collection (K1130) may be referable to *Pelobatochelys* [55], but this material was never described in the literature. A partial carapace and associated limb and girdle elements (NHMUK R8699) from Egmont Bight (Isle of Purbeck, Dorset, UK) were recently described by Pérez-García [36] as *Thalassemys* sp. and later recognized as an indeterminate species distinct from *Thalassemys hugii* [30]. This specimen is now tentatively referred to *Thalassemys bruntrutana* Püntener *et al.*, 2015 [20]. A large shell (OUMNH J.66966) from Abingdon (Oxfordshire, UK) was also recently described and assigned to *Thalassemys hugii* [20,30]. These two species of *Thalassemys* are also known from the Kimmeridgian of northwestern Switzerland.

Finally, in this study, we report the presence of *Plesiochelys etalloni* (NHMUK R3370) and *Tropidemys langii* (NHMUK OR44178b, NHMUK OR45920, NHMUK OR45921 and NHMUK R2733) in the Kimmeridge Clay turtle assemblage. The occurrence of the latter species was proposed by Lydekker [25], but recently considered inconclusive by Pérez-García [29].

### 4.4 Palaeobiogeographic implications

Plesiochelyids, thalassemydids and eurysternids are found in many Late Jurassic localities throughout western Europe, but their palaeobiogeographic distribution remains poorly understood. This is mainly the result of the lack of global (taxonomic and morphological) revision of these forms since their initial description in the nineteenth century. At that time, authors described a plethora of regional species notably in Switzerland, England, France and Germany. This material, especially the shell remains, attracted very little attention during the twentieth century. Fortunately, the interest for these coastal marine turtles was renewed in recent years [13,17,19,20,29,30,36,45]. Recent studies notably revealed how two thalassemydids (*Thalassemys hugii* and *Thalassemys bruntrutana*) were present both in Switzerland and southern England during the Kimmeridgian [20,30].

Prior to this study, no plesiochelyid species had been clearly identified outside its original area of description. *Craspedochelys picteti* Rütimeyer, 1873 [14], *Plesiochelys planiceps*, *Portlandemys mcdowelli* and *Portlandemys gracilis* are only known from their respective type locality [13,17]. *Plesiochelys etalloni* was only identified from localities within the Jura Mountains [45]. *Craspedochelys jaccardi* from the Kimmeridgian of the Swiss Jura Mountains was potentially identified in the Kimmeridgian of Murat (Department of Lot, France) [6], but this remains to be confirmed. Finally, remains referable to the genus *Tropidemys* had been signalled in many parts of Europe [29,56,57], but the species *Tropidemys langii* had only been clearly recognized from its original area of description, that is the Swiss Jura Mountains (Solothurn and Porrentruy) [14,15,19]. *Tropidemys seebachi* is the only minor exception since it is known from the region of Hannover (Lower Saxony, Germany) and from Wattendorf (Bavaria, Germany) [39,40,58].

**Figure 5.**
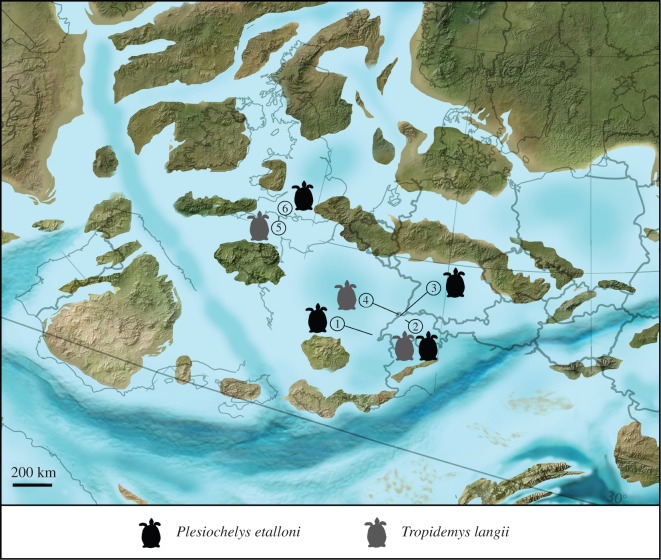
Palaeobiogeographic distribution of *Plesiochelys etalloni* (black) and *Tropidemys langii* (dark grey). Localities: (1) Moirans-en-Montagne, Department of Jura, France (type locality of *Plesiochelys etalloni*); (2) Solothurn, Canton of Solothurn, Switzerland (type locality of *Tropidemys langii*); (3) Glovelier, Canton of Jura, Switzerland; (4) Porrentruy, Canton of Jura, Switzerland; (5) Weymouth, Dorset, UK; (6) unknown locality (specimen NHMUK R3370). Palaeogeographic map from Ron Blakey, Colorado Plateau Geosystems, Arizona, USA (http://cpgeosystems.com/paleomaps.html).

The report of remains clearly referable to both *Plesiochelys etalloni* and *Tropidemys langii* in the Kimmeridge Clay Formation of southern England is therefore of major importance for the palaeobiogeograhy of plesiochelyid turtles. This is the first time, to the best of our knowledge, that the presence of these species is confirmed outside the Jura Mountains. It shows that, like thalassemydids [20,30], some plesiochelyids had a wide European distribution during the Kimmeridgian and were able to cross relatively large extents of water (figure 5). Because plesiochelyids and thalassemydids are coastal marine turtles that inhabit relatively open environments, the wide distribution of these turtles at the European scale is certainly not a surprise. In fact, we also expect more of these coastal marine turtles to have a broader distribution than currently known.

## 5. Conclusion

In this study, we report for the first time, to the best of our knowledge, the presence of the plesiochelyid turtle *Plesiochelys etalloni* from the Kimmeridgian of southern England. This species, otherwise known from the Kimmeridgian and possibly early Tithonian of the Jura Mountains (Switzerland and France), is represented by a partial basicranium from an unknown locality (NHMUK R3370). This specimen is remarkable by its robustness, but it is uncertain whether this is related to ontogeny, geographical and/or temporal variations, dimorphism or abnormal development.

Herein, we also reaffirm the initial conclusions of Lydekker [25]: several specimens from the Kimmeridge Clay of Weymouth (Dorset) are clearly referable to the plesiochelyid *Tropidemys langii*. This contradicts a recent study that concluded that this species was identified with certainty only from Switzerland [29].

This is the first time, to the best of our knowledge, that these species are conclusively signalled outside their original area of description. These results show that some plesiochelyids at least had a broader palaeobiogeographic distribution than previously thought, which should not be considered surprising for large coastal marine turtles living in the European Jurassic sea. Similar results were recently obtained for thalassemydids [20,30]. As our knowledge about these turtles progresses and historical collections are revised, we expect more plesiochelyids, thalassemydids and, possibly, eurysternids to have broader palaeobiogeographic distributions.

## Supplementary Material

File S1: 3D reconstruction of the basicranium NHMUK R3370

## Data accessibility

Three-dimensional PDF (NHMUK R3370; electronic supplementary material, file S1): figshare (http://dx.doi.org/10.6084/m9.figshare.1424890). Scaled and textured high-resolution three-dimensional mesh (NHMUK R3370): figshare (http://dx.doi.org/10.6084/m9.figshare.1513784).
