# Supplementary material for: First report of Plesiochelys etalloni and Tropidemys langii from the Late Jurassic of the UK and the palaeobiogeography of plesiochelyid turtles
Source: R Soc Open Sci. 2016 Jan 13;3(1):150470. doi: 10.1098/rsos.150470 (PMC4736927; doi:10.1098/rsos.150470)
Supplement: Supplementary file 1 [file rsos150470supp1.pdf]

NHMUK R3370
